# Supplementary material for: Too big to be noticed: cryptic invasion of Asian camel crickets in North American houses
Source: PeerJ. 2014 Sep 2;2:e523. doi: 10.7717/peerj.523 (PMC4157299; doi:10.7717/peerj.523)
Supplement: Appendix A — Full surveys used to generate data about the presence or absence of camel crickets associated with human homes, including (I) an open web survey and (II) a survey sent only to citizen scientist participants of a separate study (Wild Life of Our Homes) examining the microbial diversity of homes. [file peerj-02-523-s001.docx]

APPENDIX A

**I. What’s Living in Your House? Open Survey**

1. What is your home address? If you are not comfortable giving us your address, any address that corresponds to your block would do.
2. What is your email address?

The next questions are about nature observations in your house. Birders take good notes about what comes to their feeders. We’d love your notes about what comes to your crumbs.

1. What kinds of organisms have you seen living in your house. List as many as come to you…
2. Have you seen any camel crickets (also called cave crickets) in your house? Here’s [an image of a camel cricket](http://orthoptera.speciesfile.org/Common/basic/ShowImage.aspx?TaxonNameID=12024&ImageID=43690) just in case you aren’t sure what they look like.
3. Have you seen any ants in your house?
4. Check the boxes that apply if you have used the following in your house: ant bait, roach bait, bait or device to kill mice

**II. Wild Life of Our Homes Participant Survey**

1. What is your name and home address?
2. What is your preferred email address?
3. What kinds of organisms have you seen living IN your house? List as many as come to you.
4. Have you seen any camel crickets (also called cave crickets, spider crickets or even sprickets) in or around your house? Here’s [an image of a camel cricket](http://orthoptera.speciesfile.org/Common/basic/ShowImage.aspx?TaxonNameID=12024&ImageID=43690) just in case you aren’t sure what they look like!
5. Have you seen any ants in your house? Do you know what kind? If so, tell us!
6. Have you seen any cockroaches in your house?
7. Have you seen mice (or evidence of mice) in your house?
8. Check the boxes that apply if you have used the following in your house: ant bait, roach bait, bait or device to kill mice
9. Check the boxes that apply regarding the pets that live in your home: Cat(s), Dog(s), Cat(s) and Dog(s), No animal pets, Other (specify)
10. Do you have (and use) antibacterial dish or hand soap in your kitchen right now?
11. Is there pink biofilm (“soap scum”) present on a shower curtain or a bathtub in a bathroom in your home?
12. Do you do anything to favor or help certain species, species you like, in your home? Tell us about it….
